# Recent Advances in Glioma Therapy: Combining Vascular Normalization and Immune Checkpoint Blockade

**DOI:** 10.3390/cancers13153686

**Published:** 2021-07-22

**Authors:** Rachel L. Y. Ho, Ivy A. W. Ho

**Affiliations:** 1 National Neuroscience Institute, Singapore 308433, Singapore; rachel_ho@nni.com.sg; 2 Department of Physiology, National University of Singapore, Singapore 117593, Singapore; 3 Duke-NUS Medical School, Singapore 169857, Singapore

**Keywords:** glioblastoma, angiogenesis, immune microenvironment, immune checkpoint blockade

## Abstract

**Simple Summary:**

Glioblastoma is the most malignant tumor of the brain. Over the years, prognosis for patients with glioblastoma has remained dismal despite advances in medical sciences. Glioblastoma is a highly vascularized tumor; however, antiangiogenic therapy has not achieved the expected outcome. Recent promising results from immunotherapies for other cancer types such as melanoma have prompted the further investigation of combining antiangiogenic therapy with immune checkpoint blockade. This article aims to provide an overview concerning the development of a potential intervention that may enhance the efficacy of immune checkpoint blockade as glioblastoma therapy.

**Abstract:**

Glioblastoma (GBM) accounts for more than 50% of all primary malignancies of the brain. Current standard treatment regimen for GBM includes maximal surgical resection followed by radiation and adjuvant chemotherapy. However, due to the heterogeneity of the tumor cells, tumor recurrence is often inevitable. The prognosis of patients with glioma is, thus, dismal. Glioma is a highly angiogenic tumor yet immunologically cold. As such, evolving studies have focused on designing strategies that specifically target the tyrosine kinase receptors of angiokines and encourage immune infiltration. Recent promising results from immunotherapies on other cancer types have prompted further investigations of this therapy in GBM. In this article, we reviewed the pathological angiogenesis and immune reactivity in glioma, as well as its target for drug development, and we discussed future directions in glioma therapy.

## 1. Introduction

Glioblastoma (GBM), WHO grade IV tumor, is the most common and aggressive primary brain tumor in adult with a dismal prognosis of not more than 15 months. Current standard of care for GBM includes maximal surgical resection followed by radiotherapy (RT), concurrent adjuvant chemotherapy using temozolomide (TMZ), and occasionally alternating electric field therapy (TTFields) [1,2]. Unfortunately, despite these treatments, most tumors eventually develop resistance, resulting in recurrences of more aggressive tumors at the surgical sites or regions within 2–3 cm of the original tumor areas [3,4]. These resistant tumor cells arise due to the acquisition of additional mutations, silencing of tumor suppressor genes, and dampening of the DNA repair pathways, which drive the proliferation and growth of different tumor subclones with distinct phenotypic and molecular characteristics, giving rise to a heterogeneous tumor. Primary GBM can be classified into three molecular subtypes, namely, proneural, classical, and mesenchymal, on the basis of their transcriptomic profiles [5,6]. Each subtype has distinct characteristics genetically and clinically, and each responds differently to radiotherapy and chemotherapy [7]. Furthermore, intratumor heterogeneity is also affected by the microenvironment within the different regions of the tumor [8,9].

GBMs are highly angiogenic and contain areas of pseudopalisading necrosis and microvascular proliferation. Being surrounded by blood vessels and neurons, malignant glioma cells harness such structural physiology to actively migrate along the vasculature and white matter tracts to invade into regions distant from the original tumor mass [10,11,12]. Furthermore, GBM vasculature is characteristically unbalanced, with hyper-permeable areas associated with vasogenic edema, frequently intricated by necrotic areas within the tumor core [13]. This aberrantly altered vasculature, along with the resulting hypoxic and hostile microenvironment, facilitates the escape of malignant tumor cells. Due to such aberrant tumor vasculature, there is increasing resistance and limitations to the efficacy of conventional therapies and, as such, ongoing efforts have been in place to improve therapeutic outcomes.

As a fundamental process in vascular development and adult homeostasis, angiogenesis has also been increasingly implicated in pathological events including tumor progression and metabolic diseases. Alterations in the balance between anti- and proangiogenic mediators, resulting from gene aberrations and hypoxia, promotes angiogenic switch for tumor vessel growth. Such a switch from normal physiological to tumor angiogenesis is beneficial as tumor cells gain better access to oxygen and nutrients, as well as utilize the healthy lymphatic vessels to metastasize to distant tissues. Signaling regulators essential for modulating angiogenesis, such as vascular endothelial growth factor (VEGF), platelet derived-growth factor (PDGF), angiopoietin (ANGPT), and transforming growth factor-β (TGF-β), as well as the angiogenic process, have been extensively reviewed [14,15].

Such growth factors, cytokines, and chemokines secreted by glioma cells promote infiltration of cells including glioma stem cells (GSCs), endothelial cells (ECs), pericytes, reactive astrocytes, granulocytes, and immune cells, particularly microglia, macrophages, myeloid-derived suppressor cells (MDSCs), regulatory T (T_reg_) cells, and effector T cells [8,16]. These noncancerous cells define a glioma niche exerting its maximum influence on creating an immunosuppressive tumor microenvironment (TME), which is supportive of tumor growth and metastasis. Infiltrating immune cells are induced by specific environmental cues to elicit anti-inflammatory and immune escape responses. In malignant gliomas, tumor-associated microglia/macrophages (TAMs), the main component of the glioma microenvironment, crosstalk with T_reg_ cells to release proangiogenic and immune-suppressive VEGF as part of shaping an antitumor microenvironment [17]. Various transcriptomic analyses have revealed immune gene signatures, correlating to glioma pathology, treatment response, and survival benefits [18,19]. With compelling evidence of glioma-elicited immune dysfunction, targeting immune cells to reactivate a proinflammatory response is currently of high interest.

Rapid progress in cancer immunotherapy especially immune checkpoint blockade (ICB), has revolutionized the treatment of care for many solid tumor malignancies, including non-small-cell lung cancer and melanoma, which has driven the study of similar treatment regimen in glioma. A high density of intratumoral suppressive myeloid cells is present in the glioma immunological landscape. This immune subpopulation impairs T lymphocyte functions to mediate ICB resistance, and it is inversely associated with glioma patient survival outcome [20,21]. Furthermore, T-cell exhaustion is enriched within the glioma, characterized by an elevated co-expression of multiple co-inhibitory classical and alternative immune checkpoints on T-cell infiltrates [22], which are currently investigated as promising inhibitory targets. Notably, therapeutic effort has shifted emphasis to either priming immune cells to specific glioma-related antigens or regulating the TME to potentiate glioma immunity, achieved directly or indirectly via immune checkpoint inhibitors.

In this article, we focus on the angiogenic switch and its implications in pathological angiogenesis in glioma, as well as the contribution of key immune subpopulations, along with angiogenic factors underlying the pathogenesis of glioma. We appraise the combination of emerging viable strategies of antivascular and/or glioma immune microenvironment ICB interventions.

## 2. Glioma Angiogenesis

**Figure 1:**
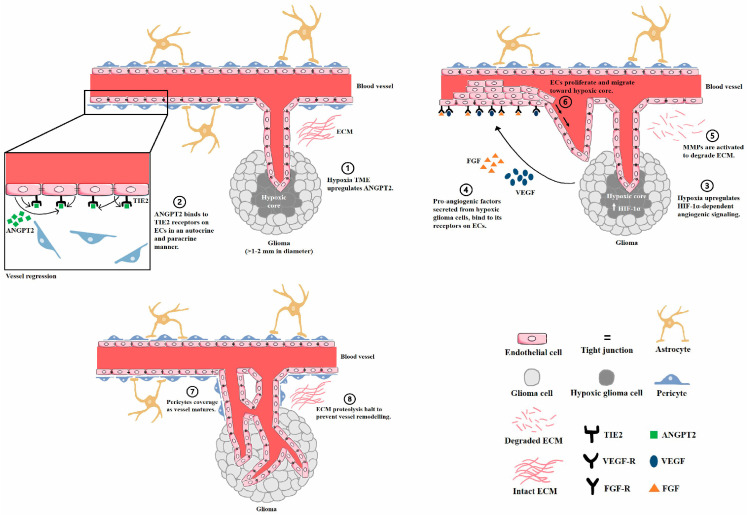
Schematic representation of glioma angiogenesis. As tumor growth within the brain parenchyma goes beyond 1–2 mm in diameter, its metabolic demands cannot be met entirely via diffusion. (1) Hypoxia then occurs to regulate angiogenic signals. (2) The activation of autocrine and paracrine ANGPT2/TIE2 signaling subsequently disrupts endothelial–mural cell interactions for vessel regression. (3,4) HIF-1α upregulation induces the transcriptional activation of proangiogenic molecules that initiate EC proliferation and migration. (5) Simultaneously, increased ANGPT2 activates MMP-2 to degrade the ECM. (6) Proteolysis of the vessel’s basement membrane facilitates the migration and proliferation of ECs toward the hypoxic tumor core. Lastly, the blood vessel wall matures, as (7) pericytes are recruited along the ECs to stabilize the blood vessel. (8) Endogenous protease inhibitors and antiangiogenic factors locally halt ECM proteolysis to hinder further vessel remodeling.

As with other solid malignancies, glioma angiogenesis is a multistep process involving (1) basement membrane and extracellular matrix (ECM) degradation, (2) EC proliferation and migration, and (3) new tumor vascular formation and organization (Figure 1). In the initial stages of glioma development, the BBB is not disrupted as the tumor mass is highly sustained by the normal brain vascular network since tumor vessels have yet to form [23]. As the glioma progresses and tumor growth within the brain parenchyma goes beyond 1–2 mm in diameter, the metabolic demands of the tumor, particularly in the core, cannot be entirely met via diffusion. Hypoxia, a hallmark of pathological angiogenesis, then occurs to upregulate proangiogenic factors and downregulate antiangiogenic signals. Activation of proangiogenic ANGPT2 and TIE2 signaling subsequently disrupts interactions between ECs and mural cells for vessel regression [24]. Furthermore, increased ANGPT2 will increase matrix metalloproteinase (MMP)-2 for the proteolysis of the ECM and vessel basement membranes [25]. This is necessary before ECs proliferate and migrate toward the hypoxic tumor cells, secreting high levels of angiogenic molecules as chemoattractants in the microenvironment. Various molecular mechanisms involving VEGF and TGF-β signaling eventually act in concert to favor hypoxia-dependent angiogenesis. Hypoxia-inducible factor (HIF)-1α expression is the major molecular basis for transcriptional activation of VEGF, a main driving factor resulting in glioma angiogenesis. Notably, HIF-1α and VEGF are highly overexpressed, especially in necrotic regions of pseudopalisades, reflecting that such expression patterns of both genes are regulated by tumor oxygenation [26]. Hypoxia-mediated upregulation of proangiogenic factor secretion by stromal and tumor cells further aggravate vascular abnormalities, thereby driving a constitutively active positive feedback loop of pathological angiogenesis. Lastly, endogenous protease inhibitors and antiangiogenic factor such as angiostatin, thrombospondin, and endostatin locally halt ECM proteolysis to hinder further vessel remodeling. Neovascularization takes place but with partial maturation as pericytes tend to detach due to persistent angiogenic stimuli for continuous vessel remodeling, resulting in immature vessels with enhanced permeability. The outcome of this neoplastic angiogenesis is an altered vasculature that is characterized by irregular branching, arteriovenous shunts and tortuous vessels, which can also lead to perfusion abnormalities [27,28]. As such, GBM has an immature neovasculature with a high leakage tendency, resulting in a loss of blood–brain barrier (BBB) integrity that is compromised both structurally and functionally.

### Perivascular Niche and Vascular Minicry in the Glioma Microenvironment

The GBM perivascular niche (PVN) consists of multiple cell types including GSCs, neural stem cells, astrocytes, ECs, pericytes, microglia, and other immune cells [29], all of which are critical for the maintenance of the cancer stem-cell state. GSCs are often found in direct close contact with the ECs, allowing the secretion of factors such as nitric oxide (NO) [30], Ephrin B2 [31], interleukin (IL)-8 [32], TGF-β, and PDGF to induce expression of stemness proteins, including CD133, SRY box transcription factors (Sox)-2, Bmi-1, and oligodendrocyte lineage transcription factor (Olig)-2 [33,34,35,36]. These interactions between GSCs and ECs encourage the invasion of GSCs, facilitating the transition from the proneural to the mesenchymal tumor subtype. In line with this, Shiraki et al. found that CD109^+^ glioma cells preferentially colocalized with CD44, a mesenchymal tumor subtype marker, to the PVN, suggesting a role of CD109^+^ cells in promoting GBM invasion [37,38].

In addition to vascular co-option and neovascularization, glioma achieves angiogenesis through vasculogenic mimicry, which is the formation of blood vessels by the tumor cells independently of ECs. Vasculogenic mimicry is often observed in the hypoxic tumor microenvironment and is characterized by an increase in HIF-1α/MMP-9/VEGF signaling [39], the activation of epithelial-mesenchymal transition (EMT)-related proteins such as Twist1 [40,41,42], and an upregulation of proinflammatory molecules such as IL-6 [43]. Vasculogenic mimicry not only sustains tumor cell growth exponentially but also encourages GSCs to form tubular networks that mimic the healthy endothelial-lined vasculature [44]. Similar to normal stem cells, GSCs are shown to have the potential to differentiate into ECs [45,46,47,48,49] or even pericytes to contribute partly to the formation of tumor vessels [50]. Using conditioned media from tumor-derived ECs, Fessler et al. showed the conversion of differentiated GBM cells to GSC-like cells via basic fibroblast growth factor (bFGF) [48]. Soda et al. showed that tumor cells may directly differentiate into CD31^+^ CD34^+^ ECs that contribute to resistance to anti-VEGF therapy [47]. Along the same vein, Wang et al. demonstrated that a subpopulation of tumor-derived EC shares the same somatic mutations as GBM cells, suggesting that the tumor EC originated from GBM cells [46]. However, endothelial transdifferentiation of GBM cells is a rare event due to the low frequency of tumor-derived endothelial cells (TECs) found within the tumor vessels [51]. The robust neovascularization yet low frequency of TECs observed can be explained by the recent findings from Dephino’s group, whereby activation of Wnt5A in TECs results in recruitment and proliferation of host ECs to promote glioma cell invasion [52]. More recently, Baisiwala and colleagues suggested that chemotherapeutic stress as a result of TMZ treatment increases HIF response in recurrent GBM, leading to the transdifferentiation of GSCs to EC [49].

In contrast to the close association of EC with tumor cells, pericytes are often found to wrap around the vessels and provide support and stability to the vasculature through a direct cell–cell contact with the EC. Tumor vessels with less pericyte coverage are, thus, less stable and more sensitive to radiation and chemotherapy [53]. Several studies have shown that GSCs give rise to vascular pericytes that support vessel function and tumor growth [13,44,50,54]. In line with these studies, the work by Cheng et al. further supported the importance of GSCs in neovasculature by showing that the GSCs develop into vascular pericyte cell fates in vitro and in a mouse xenograft model, in part through the stromal-derived factor (SDF)-1/C-X-C chemokine receptor (CXCR)-4 axis [50]. Further gene analysis found that the majority of these differentiated pericytes harbor the same genetic aberrations found in GBM, which provides valuable insights for glioma therapy. The ability of GSCs to generate ECs and pericytes allows active neovascularization at the perivascular niche to support GBM invasion and proliferation. This finding is significant as it reflects that the fate of GSCs is not only restricted to the neuroepithelial cell lineage.

## 3. Glioma Immune Microenvironment

Abnormalities in the tumor vasculature create an immunosuppressive microenvironment that severely impacts the proliferation, infiltration, and survival of immune cells. Regulatory immune cells including TAMs, microglia, MDSC, and T_reg_ cells are abundantly present in the GBM microenvironment, while antitumor lymphocytes are relatively absent [55,56]. TAMs and microglia occupy up to 50% of the entire brain tumor mass [9,57,58]. The percentage of immune cells present in the GBM appears to be associated with the molecular subtypes [59]. TAMs are enriched in the mesenchymal GBM and are associated with *NF1* mutation, whereas CD8^+^ and CD4^+^ tumor-infiltrating lymphocytes (TILs) are absent from the classical and IDH-mutant proneural tumor.

Macrophages are generally polarized into two main phenotypes, the classically activated M1-like and the alternatively activated M2-like cells. M1-like macrophages exhibit antitumor properties through the expression of signal transducer and activator of transcription (STAT)-1, nuclear factor kappa B (NF-κB), and the induction of T-helper (T_H_1) cytokines such as IL-12, tumor necrosis factor (TNF)-α, and interferon (IFN)-γ. By contrast, the M2-like macrophages express CD163, CD204, CD206, and STAT3 and secrete high levels of immunosuppressive cytokines including TGF-β and IL-10, which elicit a suppressive effect on cytotoxic CD8^+^ T lymphocytes, thereby favoring tumor progression [60,61]. TAMs are attracted to the GBM milieu through GBM-derived cytokines such as C–C motif chemokine ligand (CCL)-2, colony stimulating factor (CSF)-1, granulocyte macrophage-stimulating factor (GM-CSF), C–X3–C motif chemokine ligand (CX3CL1; also known as fractalkine), VEGF, MMP2, TGF-β, SDF-1, and osteopontin (OPN) [62,63,64,65,66]. OPN is secreted by GBMs, as well as GSCs, to mediate TAM infiltration into the tumor milieu through the interaction with integrin α_v_β_5_ [64,67]. Upon accumulation in the tumor site, these tumor-suppressive TAMs drive immune suppression and angiogenesis through anti-inflammatory factors such as arginase (Arg1), TGF-β, IL-10, and VEGF [68,69]. The presence of these immunosuppressive cytokines reduces major histocompatibility complex (MHC) expression and compromises the microglia’s antigen-presenting ability to elicit immune responses.

Although a high percentage of M2-polarized cells were suggested to accumulate in the GBM TME, the M1/M2 dichotomy following macrophage/microglia polarization in humans is not as distinct as in the experimental model using animal cells. In fact, the boundaries between the M1-like and M2-like macrophages are rather unclear. Comprehensive profiling of human glioma-derived TAMs suggested that the immune cells assume phenotypes along the M1–M2 continuum [70]. Similarly, in a murine CNS injury model, the macrophages were found to simultaneously present both M1 and M2 activation markers [71]. In line with this, the group of Muller et al. showed the co-expression of proinflammatory and alternatively activated genes in individual glioma cells using single-cell RNA-sequencing of GBM biopsies [72]. These findings suggested that, instead of existing as distinct populations of either M1-like or M2-like cells, TAMs transit through different cell states depending on the influence of their microenvironment. The heterogeneity of TAMs extends to the localization of these cells. Microglia-derived TAMs are enriched at the tumor periphery, while macrophages are typically found near the PVN and the tumor core [73,74,75]. The differential localization of these two populations of TAMs coincides with the expression of anti-inflammatory and proangiogenic factors such as IL-1 receptor antagonist (IL1-RN) [76] and VEGF at the tumor core, suggesting that these cells crosstalk with ECs and GSCs within the PVN to further support and amplify the expansion of tumor vasculature with irregular morphology and ECM remodeling [75].

### Crosstalk between Immune Cells and Angiogenesis

**Figure 2:**
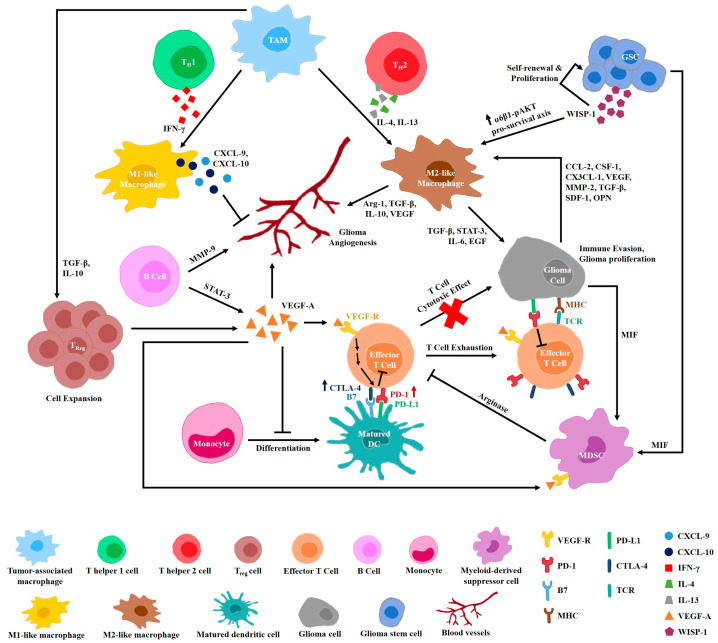
Crosstalk between immune cells and angiogenesis in glioblastoma. Glioma angiogenesis is the result of complex interactions with the immunosuppressive TME that consists of glioma cells, GSCs, and the various immune cells. M1-like and M2-like macrophages are polarized via T_H_1-derived IFN-γ and T_H_2-derived IL-4/IL-13, respectively, to mediate angiogenesis. The expansion of Treg releases VEGF-A to sustain tumor vessel growth. B cells modulate angiogenesis directly by producing STAT-3-dependent VEGF-A and MMP-9. VEGF signaling hinders DC maturation and its antigen presentation, suppresses effector T-cell functions via inhibitory immune checkpoint stimulation, and enhances MDSCs immunosuppressive activity, leading to glioma immune evasion. Macrophage migration inhibitory factor (MIF) secreted by GSCs and glioma cells acts on MDSCs-driven T-cell dysfunction. An M2-like TAMs phenotype can be further promoted by GSC-derived WISP-1 via the integrin α6β1/pAKT axis. Crosstalk between glioma cells and M2-like TAMs via various chemokines and cytokines occurs to enhance immune suppression and angiogenesis, as well as tumor cell proliferation.

TAMs, T_reg_ cells, neutrophils, and mast cells facilitate tumor sprouting angiogenesis by secreting proangiogenic molecules such as VEGF-A, IL-6, IL-8, and MMP-9 to support EC activation, proliferation, and survival [77,78]. Furthermore, circulating monocytes differentiate into TIE2-expressing TAMs to provide paracrine proangiogenic and ECM remodeling support for ECs of sprouting blood vessels [79,80,81]. Certainly, immunosuppressive M2-like TAMs are primarily enriched in tumor hypoxic regions and have proangiogenic activities in vivo. Crosstalk occurs between myeloid cells and lymphocytes to regulate tumor angiogenesis indirectly (Figure 2). While T_H_1-derived IFN-γ may trigger TAMs to elicit STAT-1-induced C-X-C motif ligand (CXCL)-9- and 10-mediated angiostatic responses, T_H_2-derived IL-4 promotes M2-like TAM activation by STAT-6 to enhance angiogenesis [82,83]. Likewise, TAM-secreted anti-inflammatory cytokines including TGF-β and IL-10 drive T_reg_ cell expansion to release VEGF-A for sustaining tumor vessel growth [17]. Moreover, B cells can modulate angiogenesis directly by producing STAT-3-dependent VEGF-A and MMP-9 or indirectly via immunoglobulin G (IgG) to trigger proangiogenic macrophage polarization upon IgG receptor activation of myeloid cells [84,85]. Thus, the intertwined relationship between hypoxia and the suppressive immune microenvironment has a crucial role in promoting tumor angiogenesis.

The VEGF family of proteins not only mediates tumor angiogenesis, but binding of VEGF to its receptors also results in the suppression of antigen-presenting cells and effector T cells, while enhancing the immune-suppressive activity of MDSC and T_regs_. Activation of VEGF signaling prevents the differentiation of monocytes into matured dendritic cells (DCs), thereby inhibiting antigen presentation, and it induces programmed death ligand 1 (PD-L1) expression in the DCs. Similarly, programmed cell death protein 1 (PD-1) and cytotoxic T-lymphocyte-associated protein 4 (CTLA-4) expression in the T cells is also upregulated by VEGF, leading to T-cell exhaustion. ANGPT2 interaction with its receptor TIE2 increases the infiltration of neutrophils and promotes its adhesion, in addition to TIE2-expressing monocytes, to the tumor endothelium [86]. On the other hand, TGF-β promotes the expansion of T_regs_ that inhibits the cytotoxic function of cytotoxic T lymphocytes (CTLs) and antigen-presenting cells, induces T-cell apoptosis, downregulates MHC expression, and skews the macrophages into an alternative-activated state [87,88]. The interplay between the immunosuppressive TME and GSCs is crucial in maintaining a niche supportive of GSCs. Recently, Wnt-induced signaling protein 1 (WISP-1) secreted by GSCs was revealed to act in both an autocrine and a paracrine manner to stimulate GSCs self-renewal and proliferation, as well as promote immune-suppressive TAM survival via the integrin α6β1/phosphorylated-AKT (pAKT) axis [89]. Even though WISP-1 knockdown had no significant effect on vessel density in GSC-derived xenografts in this study, more could be investigated in terms of its relationship with proangiogenic factors such as VEGF, which is positively regulated by WISP-1 to remodel vasculature in human osteosarcoma and oral squamous cell carcinoma. All in all, the immunosuppressive GBM microenvironment could partly be contributed by such a mode of immune cell dysfunction adapted by intracranial tumors, in which its reversal may be beneficial in re-establishing immunity against pathological angiogenesis.

## 4. Therapeutic Intervention

### 4.1. Antiangiogenic Therapy

Receptor tyrosine kinases such as VEGFR and PDGFR have been the targets for antiangiogenic therapy. Monoclonal antibodies against VEGF such as the FDA-approved bevacizumab (Avastin) are used clinically in newly diagnosed and recurrent GBM [90,91], metastatic breast cancer [92], and metastatic colorectal cancer [93]. This humanized anti-VEGF antibody specifically binds to VEGF and blocks its interaction with VEGFR [94], hindering VEGF from eliciting its proangiogenic effect and eventually resulting in tumor starvation and growth inhibition. A meta-analysis of four studies of randomized phase II clinical trials for both newly diagnosed and recurrent GBMs reflected a significant improvement in progression-free survival (PFS) but not overall survival (OS) when combined with chemotherapy [90]. Furthermore, the use of bevacizumab, either alone or in combination, had a better outcome in terms of PFS and OS in recurrent GBM than in primary GBM settings [95]. It is important to consider that the tumor vascular network is heterogeneous, with varying sensitivity to VEGF-targeted therapy. This difference in treatment susceptibility could likely be attributable to nascent vessels being VEGF-dependent while mature tumor vessels have lost this dependence; with the basement membrane and pericyte coverage, they become resistant to VEGF inhibition [96]. Indeed, bevacizumab was found to suppress new tumor vessel growth, but its efficacy is much lower against pre-existing tumor vasculature [96]. Paradoxically, prolonged treatment with bevacizumab often results in tumor hypoxia that adversely induces VEGF expression, leading to increased tumor neovascularization and vessel leakiness and, thus, resulting in a shift of the hypoxic TME to a predominantly infiltrative phenotype [97].

In addition to bevacizumab, several tyrosine kinase inhibitors such as sunitinib, sorafenib, imatinib, and galunisertib (LY2157299) were investigated for their ability to inhibit PDGFR and TGF-β signaling pathways in the context of GBM. Unfortunately, sunitinib, sorafenib, and imatinib were found to be ineffective as a monotherapy for GBM treatment [98,99,100]. Similarly, galunisertib, an oral small-molecule inhibitor against TGF-β receptor type I, failed to improve the OS in patients with recurrent GBM when used in combination with lomustine [101]. Furthermore, in a phase IIa study of galunisertib in patients with newly diagnosed GBM, no difference in median OS was observed between the groups of patients treated with standard TMZ/RT and galunisertib/TMZ/RT [102].

During tumor angiogenesis, the intercellular junctions and ECM become aberrant due to the lack of pericyte coverage, thereby affecting immune surveillance. Integrins are heterodimeric molecules that consist of α and β subunits that interact with ECM molecules such as laminin and fibronectin. Alterations in integrin expression or its interaction with cell surface adhesion proteins have been shown to relate to tumor invasion and angiogenesis [103,104,105]. Integrins play a vital role in immune cell transmigration and trafficking from the endothelium into the tissues. In addition, integrins interact with adhesion molecules to facilitate antigen presentation and formation of immunological synapses that are essential for immune cell activation (reviewed by [106]). However, targeting integrin is challenging due to its switching expression between different subsets when bound to different adhesion molecules. Nevertheless, attempts have been made to design peptidic and nonpeptidic integrin antagonists possessing different binding properties. Of note, GLPG0187 is a broad-spectrum RGD integrin receptor inhibitor that showed favorable results against glioma cells in culture, but failed to show efficacy in a phase Ib study in patients with high-grade glioma and other solid malignancies [107]. Given the dichotomous role of these angiogenic molecules in physiological and cancer development, more studies are warranted to identify a suitable therapeutic partner for combinatory approaches.

### 4.2. Immune Checkpoint Blockade

ICB involves the administration of specific antibodies such as anti-PD-1 and anti-CTLA-4, designed to interfere with the binding of ligands to the checkpoint molecules, thereby preserving T-cell activated states. ICB using inhibitors (anti-PD-1, anti-CTLA-4, anti-lymphocyte activation gene (LAG)-3, anti-T-cell immunoglobulin and ITM domain (TIGIT), anti-T-cell immunoglobulin and mucin-domain containing (TIM)-3, etc.) have shown promising outcomes in preclinical studies in a mouse tumor model in many cancer types including GBM [19,108,109,110,111,112]. In clinical studies, ICB results in activation and infiltration of CD8^+^ CTLs and increased IFNγ-associated response [113,114]. Due to its success in improving the OS and prolonged therapeutic responses in a large cohort of patients, ICB is now part of the treatment regimen for numerous cancers such as melanoma, lung cancer [115,116], and metastatic disease to the brain [117,118].

#### 4.2.1. PD-1/PD-L1 Axis

PD-1, a member of the CD28 family, is constitutively expressed on activated T cells, B cells, DCs, and macrophages, while PD-L1 is often expressed on tumor cells and upregulated due to the host cell immune response, loss of PTEN, and enhanced anaplastic lymphoma kinase (ALK) signaling [119,120,121]. PD-L1 is a target of HIF-1α [122], and upregulation of PD-L1 on tumor cells in the hypoxic tumor core interacts with PD-1 on activated T-cells, leading to T-cell anergy and exhaustion or even apoptosis. Despite the promising results of immune checkpoint blockade on other cancer types including metastatic disease to the brain [115,116,117,118], immune checkpoint monotherapy targeting the PD-1/PD-L1 axis has limited success in recurrent GBM (Checkmate-143 trial; NCT02017717) [19,123]. Whether anti-PD-1 therapy is beneficial for newly diagnosed GBM is currently being investigated in two randomized phase III clinical trials, CheckMate-498 and CheckMate-548 trials (NCT02617589 and NCT02667587), as well as the phase II PERGOLA trial (NCT03899857). CheckMate-498 is evaluating the efficacy of nivolumab versus TMZ, both with concurrent radiotherapy, in patients with newly diagnosed GBM with unmethylated O^6^-methylguanine-DNA methyltransferase (MGMT), while the CheckMate-548 trial (NCT02667587) is assessing the combination treatment of nivolumab with standard radiotherapy and TMZ followed by adjuvant TMZ with nivolumab in newly diagnosed GBM with methylated MGMT promoter. On the other hand, PERGOLA is investigating the efficacy of the safety of pembrolizumab to a standard treatment regimen.

#### 4.2.2. CTLA-4/CD28/B7 Axis

CTLA-4, also known as CD152, is a structural homolog of the costimulatory receptor CD28 and, binds to the same partners CD80 (B7-1) and CD86 (B7-2) [124]. Unlike the CD28/B7 interaction which produces a stimulatory signal, the binding of CTLA-4 to B7 promotes T-cell anergy [125]. However, contrary to the PD-1/PD-L1 axis that suppresses the existing immune response, CTLA-4 signaling inhibits the initial phase of immune inactivation [126]. In preclinical studies, the blockade of CTLA-4 led to a 1.5–2-fold increase in T-cell proliferation, enhanced IL-2 production [127], and depleted T_reg_ in the tumor microenvironment [128]. Ipilimumab, a human IgG monoclonal antibody that is specific for CTLA-4, induced CD28 expression, which amplified T-cell responses. In the preclinical model of GBM, ipilimumab treatment promoted T-cell activation and proliferation, resulting in shrinkage of the tumor, and prolonged the survival of tumor-bearing mice [18,19,129,130]. In the clinical setting, intratumoral or intracavity administration of ipilimumab in combination with nivolumab was shown to be safe in patients with recurrent GBM (NCT03233152) [131]. A phase II/III study is currently underway to evaluate the combination usage of ipilimumab with nivolumab plus radiation therapy compared to the Stupp protocol for newly diagnosed MGMT unmethylated GBM (NCT04396860).

#### 4.2.3. LAG-3

LAG-3, also known as CD223, an immunoglobulin receptor protein, is found on the surface of DCs, effector T cells, T_reg_ cells, B cells, and natural killer (NK) cells [132,133,134]. LAG-3 has been suggested to bind preferentially to stable complexes of peptides and MHC class II (pMHCII) on antigen-presenting cells [135]. The selective binding enables LAG-3 to inhibit the activation of T cells that are responsive to stable pMHCII only, thereby negatively regulating antitumor immune responses [135]. Furthermore, LAG-3-expressing T_reg_ produced elevated levels of TGF-β and IL-10, influencing the TME to be more immunosuppressive [136]. In human GBM samples, LAG-3 is expressed on tumor-infiltrating immune cells, particularly in up to 30% of CD8^+^ T cells, and it is a marker of T-cell exhaustion [22,137]. Interestingly, CD8^+^ cells that only express LAG-3 are rare, and LAG-3 expression is frequently found in TILs that co-express PD-1, suggesting their concerted role in modulating T-cell dysfunction [22]. Based on these findings, simultaneous blockade of LAG-3 and PD-1 has been employed for cancer therapy [110,138,139,140]. In a preclinical mouse model of human GBM, Harris-Bookman and colleagues showed that depletion or inhibition of LAG-3 markedly improved the survival of GBM-bearing mice treated with anti-PD-1 treatment, presumably through increased production of IFN-γ [110]. Currently, a phase I clinical trial (NCT02658981) is assessing the safety and dosage of anti-LAG-3 monoclonal antibody BMS-986016 alone or in combination with nivolumab to overcome PD-1 resistance in recurrent GBM.

#### 4.2.4. TIGIT

TIGIT is a co-inhibitory immunoreceptor that is expressed on T_regs_, activated CD4^+^ and CD8^+^ T cells, and NK cells [141,142,143,144]. It binds to CD155 (poliovirus receptor) with higher affinity than its co-stimulatory ligand CD226 and inhibits the activation of immune cells [145,146]. Anti-PD-1 and anti-TIGIT dual immune checkpoint treatment in murine intracranial tumors has synergistic effects in enhancing antitumor functions of effector T cells and decreasing suppressive tumor-infiltrating DCs and T_regs_, thereby conferring significant survival benefit [111]. Of note is that the TME is restored to a more proinflammatory state as cytokines such as TNF-α and IFN-γ are increasingly released by functional CD4^+^ and CD8^+^ T cells. Furthermore, glioma tumor rechallenge revealed the establishment of immunological memory in tumor-free long-term survivors following dual treatment, thereby sustaining its prolonged survival of 90 days in contrast to the no-treatment control group which had 21 days median survival post rechallenge [111]. Human GBM sample analysis revealed that glioma tumor cells highly expressed CD155, and a substantially higher percentage of tumor-infiltrating lymphocytes were TIGIT-positive [22,147]. This provides the basis for the CD155/TIGIT axis as a potential immune checkpoint target for GBM treatment. While there are currently no anti-TIGIT clinical trials in GBM, some agents have been examined in other cancers. The safety and pharmacological aspects of TIGIT immunoregulators ASP8374 and BMS-986207 are being assessed in ongoing interventional phase Ib trial (NCT03260322) with anti-PD-1 pembrolizumab and a first-in-human phase I/IIa trial (NCT02913313) with nivolumab in locally advanced or metastatic solid tumor malignancies. Such PD-1/TIGIT blockade may shed some light as a more efficacious immunotherapy in glioma tumorigenesis.

#### 4.2.5. TIM-3

An immunoregulatory membrane protein, TIM-3, is widely present on innate immune cells, T_regs_, and T lymphocytes, particularly on CD4^+^ T_H_1 effector cells and CD8^+^ cytotoxic T lymphocytes [148]. TIM-3 has been increasingly implicated as a marker for exhausted T cells and is a vital immune checkpoint in tumor-induced immunosuppression [149]. In tumors such as non-small-cell lung cancer, clear-cell carcinoma, and hepatocellular carcinoma, TIM-3 is not only found on the CD4^+^ and CD8^+^ TILs, but also expressed on the tumor cells [150,151,152]. Tumor-derived galectin-9 has been shown to bind to TIM-3^+^ CD8^+^ TILs to induce T=cell apoptosis, leading to suppression of cancer immunity [153]. In GBM, TIM-3 expression is elevated on glioma cells and specifically enriched in GSCs [154]. Interestingly, analysis of both TIM-3 expression and MGMT promoter methylation status provides insightful prognostic value for GBM patients. A high level of TIM-3 together with unmethylated MGMT promoter is associated with worse clinical prognosis and vice versa [155]. Furthermore, effector T lymphocytes in the peripheral blood and tumor-infiltrating T cells have enhanced TIM-3, rendering a more suppressive glioma microenvironment [156,157]. By contrast, using a mouse glioma model, Kim and colleagues showed that TIM-3 expression in tumor infiltrating CD11b^+^ CD45^mild^ microglia cells was downregulated in response to secretory factors from the brain tumor [157], suggesting that TIM-3 function is context-dependent. While there are limited preclinical GBM data on TIM-3 blockade, Kim’s group demonstrated a novel triple glioma therapy that combined dual TIM-3 and PD-1 blockade with stereotactic radiosurgery, showing a significant improvement in OS [112]. Mice that received the triple therapy demonstrated an improved glioma microenvironment immuno-active profile with increased immune cell infiltration and activity, as well as significantly extended lifespan with durable immune memory [112]. This triple therapeutic strategy is of translational relevance as a phase I trial (NCT03961971) for recurrent GBM patients in evaluating the safety of stereotactic radiosurgery, spartalizumab (anti-PD-1), and MBG453 (anti-TIM-3) combination treatment is underway. Table 1 summarizes the data from reported clinical trials in this review, in newly diagnosed and recurrent GBM patients.

### 4.3. Strategies to Increase Treatment Efficacy

In the decade following its approval, ICB has emerged as one of the most promising cancer therapies. These inhibitors improved the survival of patients with GBM, melanoma, and non-small-cell lung cancer, to name a few. However, the overall response rate for many cancer types is still modest [169] and GBM is among the tumors that are refractory to immunotherapy due to drug resistance. Thus, there is an unmet crucial necessity to improve the response rate by coupling ICB with other therapeutic modalities.

#### 4.3.1. Vascular Normalization Increases T-Cell Infiltration

GBM is considered a “cold tumor” due to the substantially lower level of immune infiltrate and lack of or low response to immunostimulatory treatment strategies, partly due to hypoperfusion in the tumor hypoxic region that affects the infiltration, proliferation, and function of immune cells. A less hypoxic and functional vasculature is a pre-requisite for leukocyte migration, as well as efficient drug delivery [170]. Vascular normalization converts the immunosuppressive tumor microenvironment into an immune-stimulatory one by reducing hypoxia and ameliorating acidosis. Indeed, several preclinical studies have revealed that vessel normalization improves T-cell tumor infiltration in antiangiogenic therapy [171,172,173,174]. Using various gene knockout models of breast cancer, Tian and colleagues showed that CD4^+^ T_H_1 cells are crucial in vessel normalization. These activated T_H_1 cells secrete IFN-γ, resulting in increased expression of endothelial adhesion molecules such as intercellular adhesion molecule (ICAM) and selectin E (SELE), which subsequently leads to enhance immune infiltration [175] and stimulates pericyte recruitment through increased CXCL-9, CXCL-10, and CXCL-11 expression [171,176]. In breast cancer, a low-dose anti-VEGFR antibody (DC101) treatment normalized tumor vasculature by reconditioning the tumor vessels for improved perfusion, which are homogeneously distributed within the tumor in vivo [177]. Consequently, significantly more CD8^+^ T cells infiltrated into the DC101-treated tumors compared with IgG control. This active immune infiltration is further stimulated by CXCL9 secreted from M1-like TAMs polarized by reduced hypoxia as vessels become better perfused [177]. In line with these data, the trafficking of activated T cells is also enhanced in the regulator of G-protein signaling-5 (Rgs5)-deficient pancreatic neuroendocrine tumors. Rgs5, a modulator of vascular survival, is upregulated in abnormal tumor vasculature, and its loss results in vessel remodeling. As a result of *Rgs5* deficiency in angiogenic vessels, CD4^+^ and CD8^+^ T cells massively influx into the tumors and greatly prolong mice survival [178]. A genetic mouse study of pancreatic cancer using haplodeficient oxygen sensor prolyl hydroxylase domain proteins (Phd)2 (*Phd2+/−*) model and *Phd2Cre/+* mice model harboring conditional *Phd2* haplodeficiency in ECs reported normalization of endothelial lining and vascular maturation in vivo [179]. This tumor vessel reshaping provides survival benefits due to improved tumor oxygenation and perfusion, as well as reduced metastatic gene expression. In both mouse models, while tumor growth was not markedly reduced, vessels were more stable and EC barrier was re-established to improve oxygen supply. Interestingly, endothelial normalization by *Phd2* haplodeficiency to modulate vessel shape, instead of altering vessel density, is sufficient to trigger a shift toward reduced tumor malignancy. This reflects the potential of PHD2 inhibitors as antivascular agents in pathological angiogenesis, especially for solid tumors where vessel oxygenation is impaired due to hypoxia.

#### 4.3.2. Combining ICB with Antiangiogenic Molecules to Increase Treatment Efficacy

As mentioned previously, the overall immunosuppressive environment may contribute to the dismal response rate to ICBs. To condition an immune-stimulating microenvironment, a two-pronged antivascular approach combining vascular normalization with ICB has progressively become the focus. The benefit of using this two-pronged approach has been investigated in several cancer types and has been approved as the first-line treatment for advanced renal cell carcinoma (KEYNOTE-426) [180].

In the preclinical metastatic melanoma setting, combination with immunotherapy was shown to overcome resistance to anti-VEGF treatment as exemplified by tumor regression upon anti-PD-1 treatment, which elicits humoral immune responses by inducing anti-ANGPT2 serum IgG against ANGPT-2-derived bevacizumab resistance [181]. Furthermore, anti-PD-L1 therapy maintained and enhanced vessel normalization during sorafenib (anti-VEGFR2 antibody) treatment in breast and pancreatic neuroendocrine tumor murine models [182]. More recently, the groups of Plate and Reiss showed that blockade of VEGF, ANGPT2, and PD-1 extended the survival of GBM-bearing mice in comparison to anti-VEGF and anti-ANGPT2 alone [183]. The triple therapy increased the percentage of CD8^+^ CTLs in the tumor but decreased CD4^+^/FoxP3^+^ T_H_ cells. When CD8 cells were depleted, the efficacy of the triple therapy diminished, thus indicating the crucial contribution of the CD8^+^ T cells in this treatment strategy, a finding that is in line with that observed in breast cancer models [171].

Another strategy makes use of the tumor vasculature normalization aspect of the tumor necrosis factor superfamily member 14 (TNFSF14/CD258/LIGHT)/lymphotoxin-β receptor (LTβR) signaling axis coupled with its intrinsic ability to turn a “cold” tumor “hot”. LIGHT is expressed on activated T cells, NK cells, and immature DCs [184,185], while LTβR is found on the surface of epithelial cells, stromal cells, immature DCs, and myeloid cells [186]. LIGHT/LTβR signaling normalizes tumor vasculature via LTβR-dependent ICAM, vascular cell adhesion protein (VCAM), and smooth muscle actin (SMA) expression, and it facilitates infiltration of immune effector cells. Using a vascular targeting peptide (VTP)-linked LIGHT fusion protein approach, He et al. showed that delivery of this fusion protein ameliorated tumor perfusion and alleviated tumor hypoxia, limiting the recruitment of M2-like immune cells [187]. The use of LIGHT–VTP sensitizes angiogenic glioma by promoting mature pericyte switch for vasculature normalization and facilitating the induction of high endothelial venules (HEVs) that increased cytotoxic T-cell tumor infiltration. HEVs are venous structures that facilitate the trafficking of T cells. They are typically found in secondary lymphoid organs such as lymph nodes [188]. HEV was also detected in tumors and has been suggested to correlate with a good prognosis and patient survival [189,190,191,192,193]. Along the same line, Allen et al. found that LTβR activation during antiangiogenic and anti-PD-L1 therapy changed approximately 15% of tumor vessels into HEVs, which correlated with a 10-fold higher number of granzyme B-activated CD8^+^ cells in a preclinical mouse model of GBM [182]. These results suggested that activating HEV formation may be one avenue to overcome antivascular resistance in GBM treatment, and this encourages further exploration to achieve a more efficacious translation output.

While combination treatments are increasingly attractive as a means to suppress disease progression, their use in the clinics, especially for GBM, has yet to be established. Phase II clinical trials combining bevacizumab and pembrolizumab treatment in recurrent GBM compared to pembrolizumab alone is of limited benefit, although the PFS for the combination group was favorable when compared to the pembrolizumab monotherapy: 26% (95% CI:16.3, 41.5) and 6.7% (95% CI: 1.7, 25.4), respectively (NCT02337491) [161]. Patients treated with the monotherapy exhibited slightly better median OS than the combination therapy (10.3 months vs. 8.8 months, respectively). More importantly, the investigators failed to observe a correlation among PD-L1 expression, TIL infiltration, and immune activation gene expression profile (GEP) with the OS. Similarly, the combination usage of axitinib (VEGFR1-3 inhibitor) and avelumab (anti-PD-L1) in unselected patients with recurrent GBM (NCT03291314) [162] failed to meet the expected outcome despite being approved as a first-line treatment strategy for advanced RCC [194].

### 4.4. Challenges to ICB in GBM

#### 4.4.1. Discordance between Preclinical and Clinical Study Settings

Despite the promising results observed in the preclinical setting, unfortunately, patient clinical trials could not reproduce the high therapeutic efficacy. The discrepancy between the preclinical and clinical results is largely due to the study design. Preclinical GBM mouse models most often use the GL261 mouse glioma cells or genetically engineered mouse model (GEMM) with knockout or mutation in key tumor suppressor or enhancer genes. These models do not accurately phenocopy the complexity of human GBM, especially the heterogeneous tumor microenvironment. In particular, the GL261 mouse glioma cells are moderately immunogenic and express a high level of PD-L1, which is in contrast to human GBM [195]. Although the complexity of the human immunobiological system can be partially restored with the use of a humanized mouse model, whereby the tumor and immune system are human patient-specific [196], the cost of using a humanized mouse model is prohibitive, and the rate of tumor growth is generally much slower than the mouse xenograft model. It is important to bear in mind that, due to a lack of a suitable preclinical mouse model that closely mimics the human GBM, initiation of clinical trials evaluating the efficacy of ICB is most often based on the promising clinical data obtained from other cancer types such as RCC, which is highly angiogenic and immune-reactive.

#### 4.4.2. “Windows” of Opportunity

Several factors may contribute to the lack of efficacy of ICB and antiangiogenic combination therapy in the clinical setting. The first factor is insufficient knowledge about the window of vascular normalization and checkpoint inhibitor, which is dependent on the dosing regimen and duration of agents administered. Pruning of immature tumor vessels due to excessive angiogenesis has to be minimal as it impedes perfusion and reverts the TME to an immunosuppressive phenotype. More importantly, this hypoxic-driven angiogenesis will further support treatment-resistant GSCs in the heterogeneous GBM tumor and promote an influx of CD11b^+^ and CD68^+^ immunosuppressive myeloid cells [197], conferring a more invasive phenotype. Thus, there is an unmet need to alleviate such a paradoxical therapeutic effect, which can be achieved through a balance of low dose with an appropriate treatment period tailored to the tumor vascularity.

The timing of ICB therapy is also relevant to the success of checkpoint inhibitor-based combination therapy. In a recent study by Cloughesy et al. investigating neoadjuvant and adjuvant anti-PD-1 therapy in patients with GBM, neoadjuvant treatment was found to activate T cells and IFN-γ response within the TME [163]. The median OS of patients receiving neoadjuvant compared to adjuvant anti-PD-1 was 13.7 months versus 7.5 months (NCT02337686) [163]. Enhanced CD3^+^ CD8^+^ infiltration and increased TCR clonal diversity in the nivolumab-treated patients were also observed in another neoadjuvant nivolumab investigation in resectable GBM (NCT02550249), although no clinical benefit was observed [164].

Defining the kinetics of vessel normalization and immunobiological modifications is useful in bridging the gap on the scheduled, sequential, or simultaneous administration of antivascular drugs and ICB, to widen the window for optimal survival benefit. One needs to bear in mind that the extent of ICB and vascular normalization-induced immune activation will vary with the tumor types and perhaps the molecular subtypes. These parameters are currently under evaluation in two ongoing clinical trials (NCT02336165 and NCT03452579) in GBM. Such a study may provide a more in-depth understanding of the glioma TME post treatment.

#### 4.4.3. Biomarkers

Despite antiangiogenic and ICB combination therapy being promising, no predictive biomarkers of response and resistance are validated and available to guide drug usage. This outstanding challenge needs to be addressed to delineate usage for patients who likely benefit from such treatments due to the risks of adverse effects and high costs, thereby necessitating a concerted effort in identifying biomarkers for optimal combination. The interaction between the immune cells and the tumor cells in the TME is complicated. Hence, a combination of biomarkers will be required to reliably predict and prognosticate a patient’s response to therapy. Factors such as PD-L1 expression, tumor mutational burden, microsatellite instability, and IFN-γ gene signatures have been used as predictive biomarkers for ICB [198].

PD-L1 expression has been widely reported as a potential biomarker to predict clinical response. However, the expression of PD-L1 varies across cancer types; PD-L1 expression is higher in non-small-cell lung carcinoma than in melanoma. In GBM, PD-L1 expression was neither predictive nor prognostic in a study evaluating 135 newly diagnosed and recurrent samples [199]. Contrary to this study, high levels of PD-L1 expression were found to be associated with worse clinical outcomes in GBM patients that underwent anti-PD-1 treatment [195,200]. It is possible that PD-L1 expression in the TME, rather than the GBM, is more indicative of a clinical response due to the expression of IFN-γ by the immune cells in the stromal microenvironment. Since the efficacy of ICB therapy is affected by tumor mutational burden, the low load of neoantigens in GBM [201,202] has to be taken into consideration. Stratification of glioma patients based on the load of neoantigens, which are specifically expressed on tumor cells due to somatic DNA mutations, has the potential in determining therapeutic efficacy. Generally, a high mutational burden is more responsive to ICB [203]. As such, strategies to identify the subsets of patients benefitting from treatment and to increase neoantigen load to turn tumors with low baseline mutational burden into immunologically “hot” are desirable for overcoming the barrier to effective treatment combining immunotherapy. Together with antiangiogenic therapy, a multimodal approach incorporating glioma neoantigen screening before treatment would be insightful as an initial step for maximal antitumor immunogenic response and clinical benefit.

Tumor inflammation-associated factors can promote tumor progression. In a small randomized phase II and biomarker study investigating anti-PD-1 plus bevacizumab for patients with recurrent GBM (*n* = 51), Nayak et al. reported that an increase in baseline placental growth factor and soluble VEGFR1 level, as well as post-therapy VEGF levels, correlated with poorer survival, suggesting a possible elevation in tumor hypoxia that leads to immunosuppression. On the contrary, PD-L1, TIL analysis, and immune activation GEP analysis did not correlate with the outcome [161]. It is plausible that ICB and antiangiogenic therapy target different cell populations in the tumor and the TME, which in turn affect the tumor–immune interactions. Thus, neither single factor is sufficient to predict the individual response to ICB combination therapy. Perhaps the concept of a cancer immunogram, whereby several parameters determined from RNA-seq or tumor and plasma analysis integrate to provide a complete immunological landscape of an individual, is more advantageous as biomarkers [204,205,206,207]. This concept remains theoretical and has yet to be evaluated in GBM.

Vessel normalization by antiangiogenics provides only a transient survival advantage ranging from weeks to months with rarely robust anticancer outcome [177,208], while ICB commonly failed at the expense of intolerable toxicities in the majority of patients. The validation of biomarkers in the future will be valuable in establishing a correlation between the molecular profile and therapeutic response. This eventually aids in the enhancement of drug delivery and durable immune responses, as well as a possible cost reduction associated with dual administration of antiangiogenic and ICB as competition among drug manufacturing companies to produce inhibitors intensifies.

## 5. Conclusions

Given the intricate crosstalk among the angiogenic, adhesion, and immune environment in GBM, the design of antiangiogenic or combination therapy requires the incorporation of these complexities for reduced resistance and increased efficacy. Comprehensive validation in more prospective randomized phase III studies is required to gain a better understanding of the microenvironmental conditions that sustain the vascularization–immunity interaction, under which antiangiogenic treatment and immune checkpoint inhibitors need to target in order to exert their combined effects on glioma TME. Many questions remained unanswered. Given the heterogeneous nature of the glioma TME [8,209], is neoadjuvant combination therapy effective against all GBM subtypes? What are the cell types that are affected by ICB and antiangiogenesis treatment? It is possible that the combination therapy targets two different populations of cells, together reducing the tumor burden. Is localized delivery of ICB and antiangiogenic treatment sufficient to activate the immunoregulatory cells in the TME? With regard to the immune landscape, how reflective are the preclinical mouse glioma models in recapitulating patients’ tumor heterogeneity? Given that different syngeneic mouse models harbor distinctly different immune phenotypes [210], this has to be taken into consideration while intervening with ICB. Not one mouse model is sufficient in translating effectively to the clinical setting, owing to GBM having different subpopulations of patients. We also need to access the baseline immune profile and immunoreactivity of the preclinical models to ICB to appraise whether the chosen model is comparable to human GBM. We believe that the mouse model has to exhibit a certain degree of ICB resistance, as in patients’ GBM, for the outcome to be more reliable and translatable. While these aspects remain a challenge, further progression in the research of angiogenesis and immune modulation will eventually shed new light on achieving a sustainable antitumor response with minimal toxicity. Addressing the aforementioned areas which are lacking may truly provide crucial insights into the tumor biology responsible for the low efficacy or failure of current antivascular or combination therapies, advancing the targeted cancer therapeutics field.

## Figures and Tables

**Table 1:** Completed and ongoing ICB clinical trials in monotherapies and in combination with antiangiogenic therapy in GBM.

Target	Treatment	Setting	Study Design	No. of Patients (*n*)	Primary Endpoint(s)	Primary Outcome	Identifier	Reference
PD-1	Nivolumab vs. Bevacizumab	R	Open-label phase III	369	OS	Primary endpoint not met	CheckMate-143 NCT02017717	[123]
PD-1	Nivolumab + RT vs. TMZ + RT	ND, unmethylated MGMT	Open-label phase III	553	OS	Primary endpoint not met	CheckMate-498 NCT02617589	[158]
PD-1	Nivolumab + TMZ + RT vs. Placebo + TMZ + RT	ND, methylated MGMT	Triple-blinded phase III	693 (targeted)	PFS, OS	Primary endpoints not met	CheckMate-548 NCT02667587	[159]
PD-1	Pembrolizumab + TMZ + RT	ND	Open-label phase II	56 (targeted)	OS	Recruiting participants	NCT03899857	
CTLA-4	Ipilimumab + Nivolumab	R	Open-label phase I	27	PFS, OS	mPFS 2.7 mo mOS 8.7 mo	NCT03233152	[131]
CTLA-4	Ipilimumab + Nivolumab + RT vs. TMZ + RT	ND, unmethylated MGMT	Open-label phase II/III	485 (targeted)	PFS, OS	Recruiting participants	NCT04396860	
LAG-3	Anti-LAG-3 mAb (BMS-986016) +/− Nivolumab	R	Open-label phase I	33	MTD	Late-onset DLT, DLT rate <33%	NCT02658981	[160]
TIGIT	Anti-TIGIT mAb (ASP8374) +/− Pembrolizumab	Advanced solid tumors	Open-label phase Ib	169	Safety and tolerability: DLT and AE	Result pending	NCT03260322	
TIGIT	Anti-TIGIT mAb (BMS-986207) +/− Nivolumab	Advanced solid tumors	Open-label phase I/IIa	130 (targeted)	AE, SAE, ORR, mDOR, PSF rate	Recruiting participants	NCT02913313	
TIM-3	Anti-TIM-3 mAb (MBG453) + spartalizumab + SRS	R	Open-label phase I	15 (targeted)	SAE	Recruiting participants	NCT03961971	
PD-1 + VEGF	Pembrolizumab +/− Bevacizumab	R	Open-label, randomized phase II	80	MTD, DLT, PFS6	MTD 200 mg/3 weeks No DLT PFS6 26% vs. 6.7%	NCT02337491	[161]
PD-L1 + VEGFR	Avelumab + Axitinib	R	Open-label phase II (2 cohorts)	54	PSF6	PSF6 22.2% vs. 18.5%	NCT03291314	[162]
PD-1	Neoadjuvant +/− adjuvant Pembrolizumab	R	Open-label, randomized phase II	35	PSF	mPFS 3.3 mo vs. 2.4 mo	NCT02337686	[163]
PD-1	Neoadjuvant Nivolumab	ND & R	Open-label phase II	30	Changes in PD-L1 percentage and expression levels by lymphocytes	Nivolumab vs. control: no significant changes in immune cells’ PD-L1 expression	NCT02550249	[164]
PD-L1	Durvalumab + RT (Cohort A)	ND, unmethylated MGMT (Cohort A)	Open-label, multi-cohort phase II	40	OS-12	mOS 15.1 mo OS-12 60%	NCT02336165	[165]
PD-L1 + VEGF	Durvalumab +/− Bevacizumab (Cohort B)	R, bevacizumab- naïve	Open-label, multi-cohort phase II	97	PFS-6	PFS-6 B1: 20%; B2: 15.2%; B3: 21.1%	NCT02336165	[166]
	Durvalumab + Bevacizumab (Cohort C)	R, bevacizumab- refractory	Open-label, multi-cohort phase II	22	OS-6	OS-6 36.4% ≥22 weeks	NCT02336165	[167]
PD-1 + VEGF	Neoadjuvant Nivolumab + low/standard Bevacizumab dosage	R	Open-label, randomized phase II	90	OS-12	OS-12 58%	NCT03452579	[168]

Abbreviations: R, recurrent; ND, newly diagnosed; MGMT, O^6^-methylguanine-DNA methyltransferase; mo, months; OS, overall survival; OS-6, OS at 6 months; OS-12, OS at 12 months; mOS, median OS; PFS, progression-free survival; mPFS, median PFS; PFS6, 6 month PFS; RT, radiotherapy; TMZ, temozolomide; mAb, monoclonal antibody; MTD, maximum tolerated dose; DLT, dose-limiting toxicity; AE, adverse event; SAE, serious AE; ORR, objective response rate; mDOR, median duration of response; SRS, stereotactic radiosurgery.
